# Supplementary material for: Identification of New Chromosomal Loci Involved in com Genes Expression and Natural Transformation in the Actinobacterial Model Organism Micrococcus luteus
Source: Genes (Basel). 2021 Aug 25;12(9):1307. doi: 10.3390/genes12091307 (PMC8467076; doi:10.3390/genes12091307)
Supplement: Supplementary file 1 [file genes-12-01307-s001.zip › genes-1336703-supplementary.pdf]

Supplementary Material

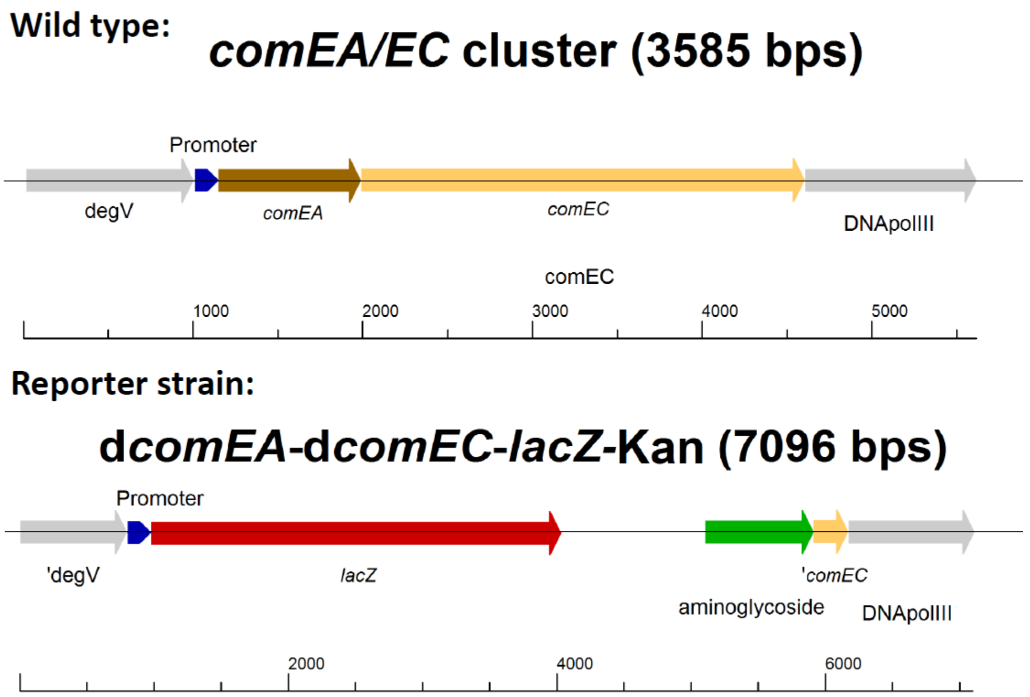

Figure S1. Chromosomal situation at *comEA/EC locus in* M. luteus trpE16 wildtype and in the Δ*comEA/EC:lacZ-Kan* reporter strain. The 3.5 kbp region of *comEA-comEC* of *M. lutues* trpE16 was replaced by a 5 kbp construct containing the complete *E. coli lacZ* gene followed by a resistance cassette conferring resistance against the aminoglycoside antibiotic kanamycin [26].

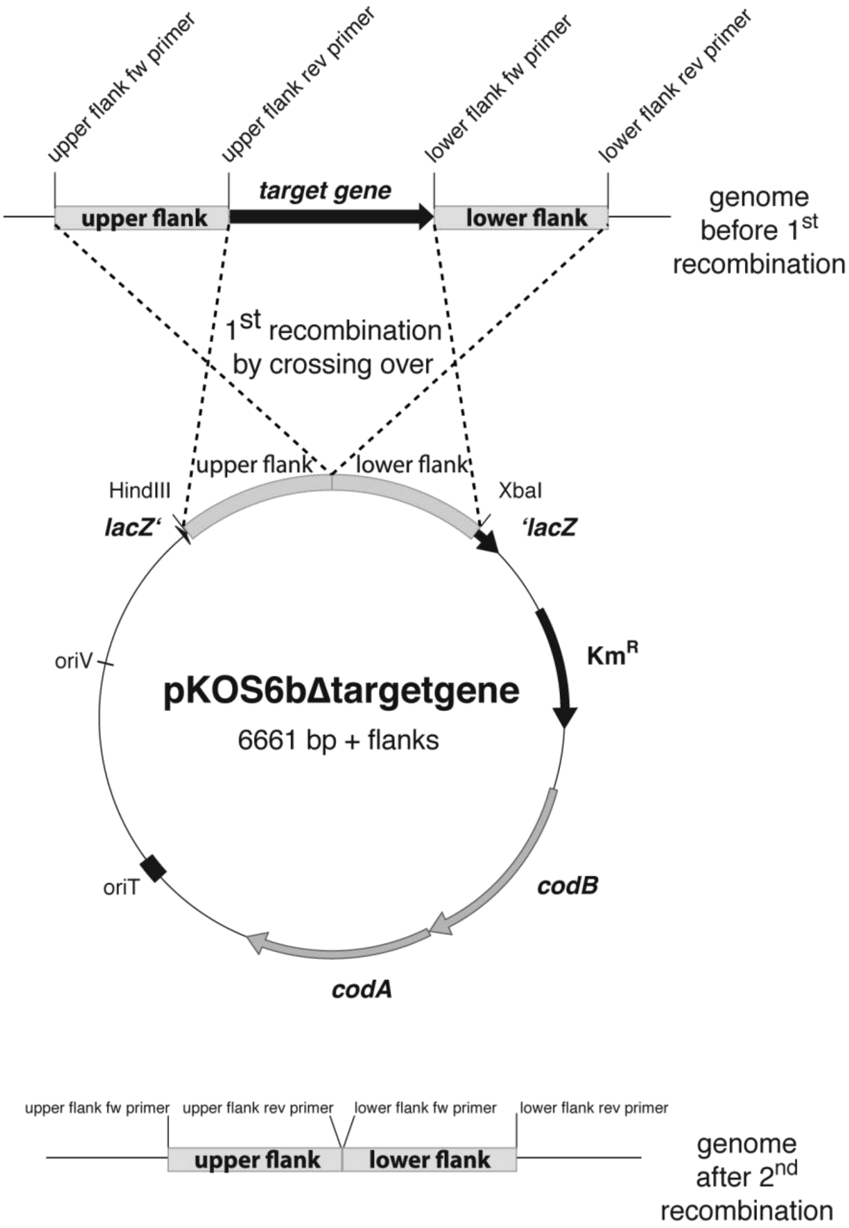

Figure S2. Vector map of pKOS6bΔtarget gene used for clean deletions and genome editing via double crossover recombination. The figure shows a general scheme with an exemplary vector. Homologous recombination occurs via ~1 kbp regions flanking the target gene or the chromosomal position to be changed. After a second recombination event there is an about 50% chance to obtain the desired modification in the genome, either the deleted locus or the new nucleotide. Labeling: origin of replication (oriV), origin of transformation (oriT), cytosine deaminase gene (*codA*), cytosine permease gene (*codB*), both restriction sites (XbaI, HindIII) found in the multiple cloning site within the *lacZ* gene and used for plasmid digestion, two representative flanks (upper, lower) and the kanamycin resistance cassette (Km^R).

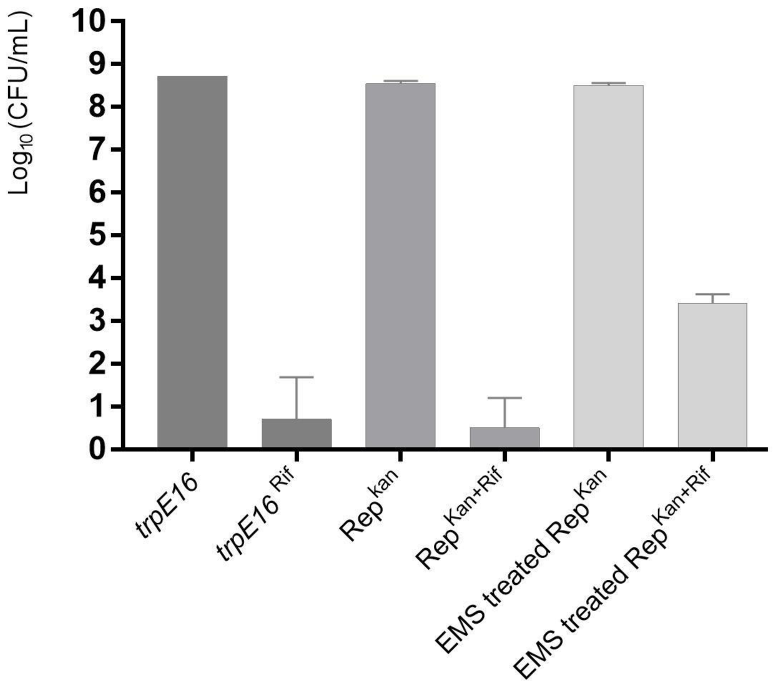

Figure S3. Log10 transformed number of rifampicin resistant colonies obtained with and without EMS treatment. Both the *trpE16* wild type and the *lacZ* reporter strain were plated on LB along with EMS-treated cells of the *lacZ* reporter strain. The medium with the *lacZ* reporter strain additionally contained kanamycin. The error bars show the standard deviation of three biological replicates. An unpaired, two-tailed Student´s test showed a significant difference between the mean values of the treated and untreated cultures ($p<0.05$, $n>3$), meaning a higher mutation rate for the EMS-treated cells.

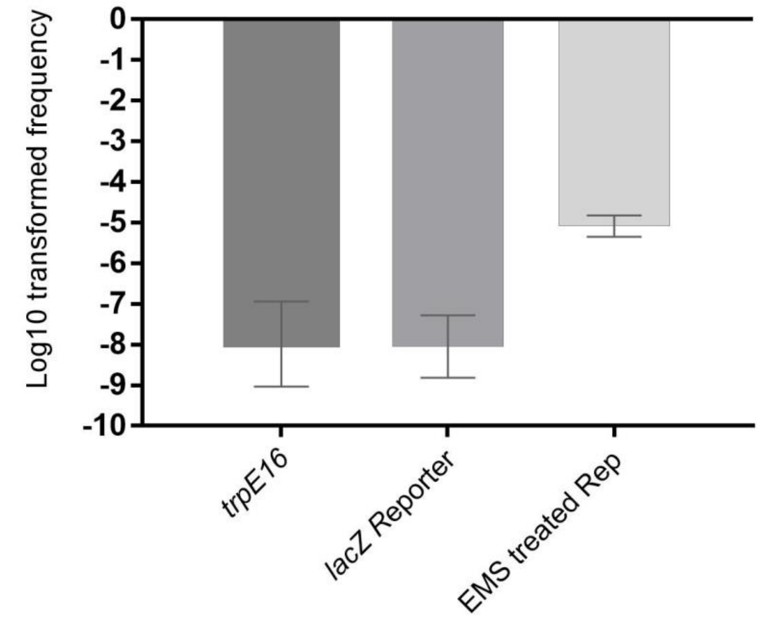

Figure S4. Frequency of rifampicin resistance appearance. Frequency of appearance of colonies of rifampicin resistant mutants arising from *trpE16* cells not treated with EMS and from *lacZ* reporter strain cells either untreated or treated with EMS. An around 3 log^{10} scale difference between the resistance frequencies of the treated *versus* the non-treated cultures. An unpaired, two-tailed Student´s test showed a significant difference between the mean values of the treated and untreated cultures ($p<0.05$, $n>3$).

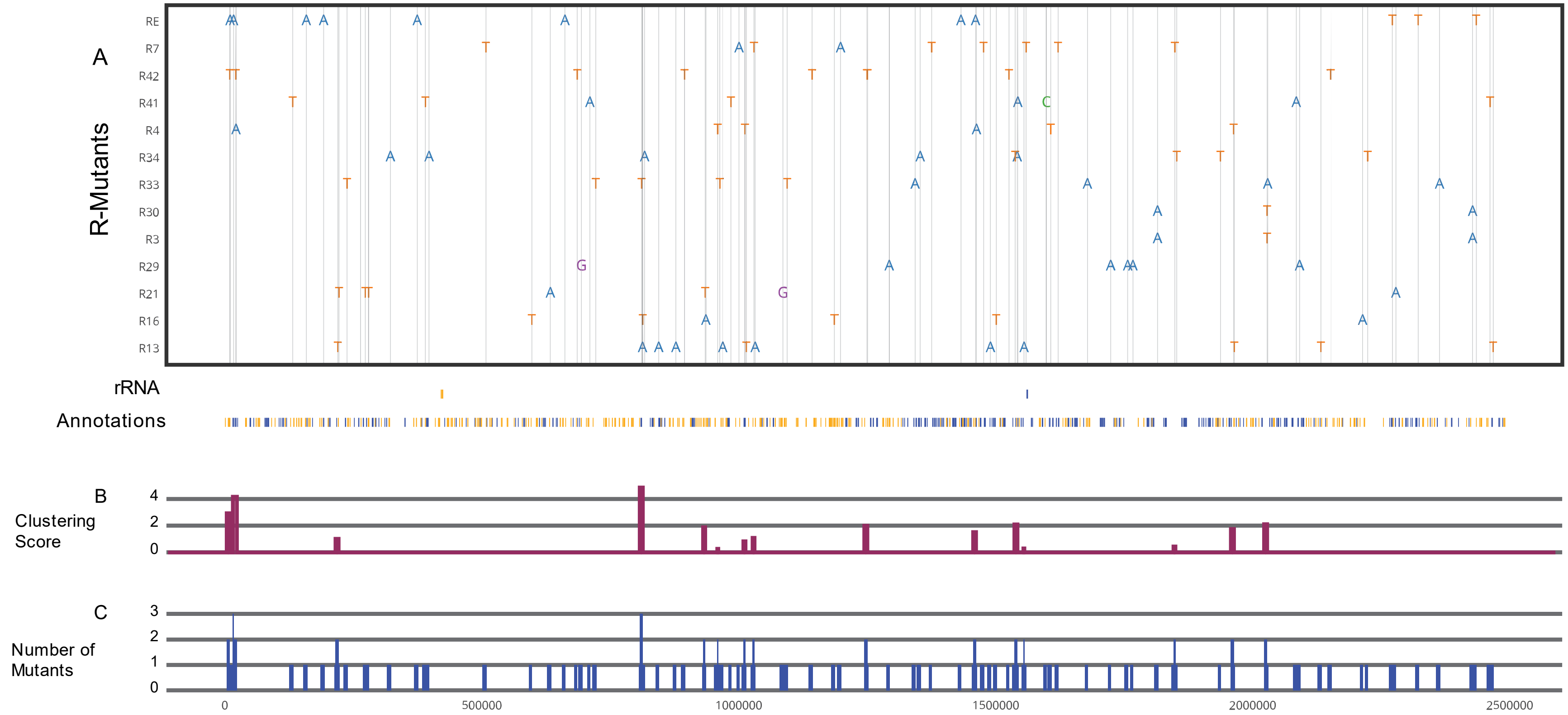

Figure S5. Distribution and clustering analysis of SNPs in R-mutants. All plots in the figure share the horizontal axis which represents the reference genome of *M. luteus* trpE16. Every SNP of each mutant is shown in the upper panel. A) All aligned R-mutants and their SNPs, along with the annotated genes affected by each mutation. B) Clustering score for each position, pointing out the most affected areas in the genome. This score is the result of a programmable sliding window function that was applied throughout the genome. The window coverage was set to 5 kbp. C) Number of mutants holding a mutation at each position in the genome. The mutations do not appear to be particularly clustered at any specific position.

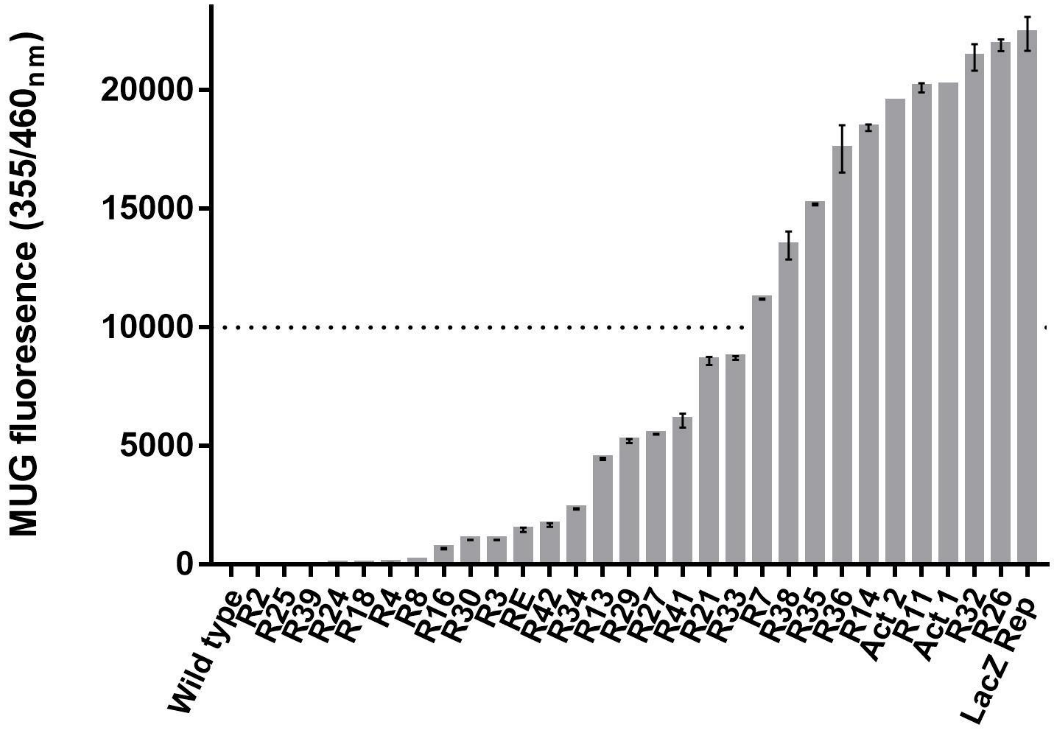

Figure S6. β-galactosidase activity assay based on the fluorescent substrate 4-methylumbelliferyl β-D-galactopyranoside. The assayed strains were cultured overnight in LB broth and were all normalized to an OD_{600} of 1.5. After lysozyme treatment (0.1 mg/mL for 10 min at 37°C), 200 µL of crude extract from each sample was mixed with the substrate to a final concentration of 250 µg/mL. A 10 min end-point measurement was made at 460 nm absorbance. Only those R-mutants which had less than half of the Δ*comEA*/*EC*:*lacZ* reporter strain activity were taken for further analysis. The *trpE16* wild type strain was used as negative control and the *lacZ* transcriptional reporter as maximum activity reference. Act1 and Act2 were two isolates with known high activity used as positive controls. All mean values and standard deviations were represented and derive from at least 3 independent repetitions.

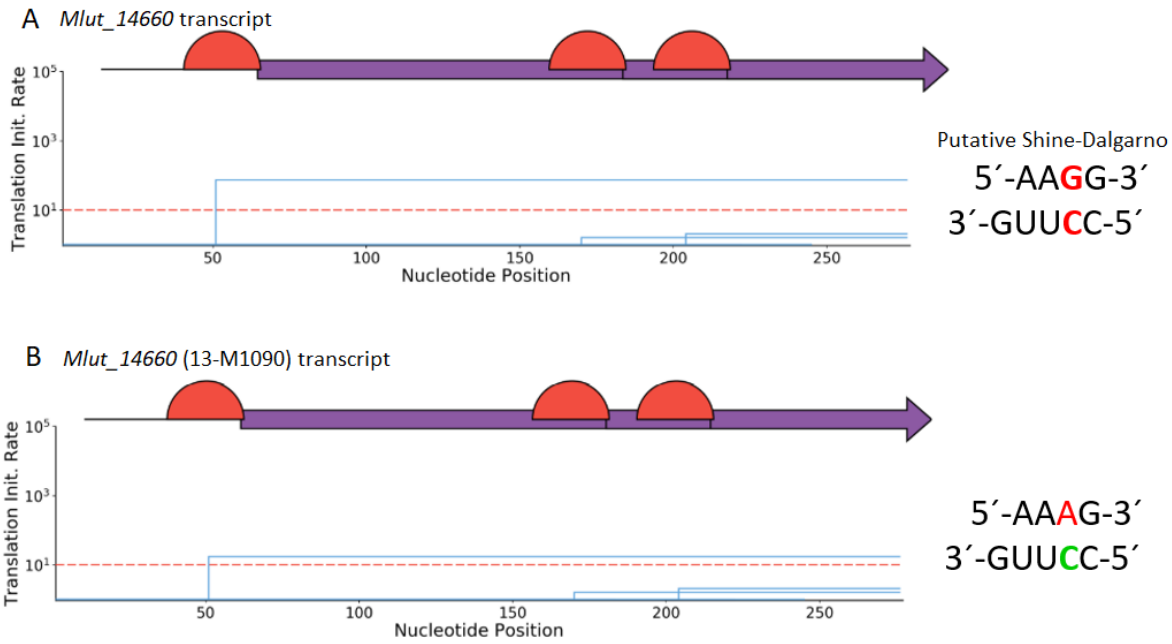

Figure S7. Translation initiation rates of *M. luteus* trpE16 and the mutant 13-M1090. Translation initiation rates for each starting codon found at the mRNA sequence of *Mlut_14660*. The RBS calculator program was used (**https://salislab.net/software/predict_rbs_calculator**) to compare the values of *M. luteus trpE16* (A) and the mutant 13-M1090 (B). A significant decrease in translation initiation is predicted after the introduction of mutation G1607117A.

R-Script for SNPs mapping and clusters determination

```r
# script to vizualise SNP data from the EMS experiment

library(tidyverse)
library(plotly)
library(data.table)
source("snp-clustering-score-function.R")

#setwd("C:/Users/Student/Dropbox/TUM Team Folder/EMS-R")
#After procesing all genomes and filtering good quality/high coverage mutations, a table with mutants, mutated positions in the genome,
# and type of mutations is crated

#First, a Data Frame with all nucleotides -ordered by mutant- that differ from the reference is created.
df <- read_delim("results.tab", delim = "\t")
df.long <- df %>% gather("strain", "mutation", M1090:`374-trpE16-out-trp`) %>%
                  mutate(mutation1 = ifelse(mutation == Reference, " ", mutation)) # crazy bug, if mutation1 is NA or "", plotly zoom doesn't

df.cons <- df.long %>% filter(mutation1 != " ") %>% group_by(POS) %>% summarise(n = n())

#A plot is created.The names of the mutants on the Y axe and all the positions of the genome on the X axe, so each mutated position shows up
# by presenting the SNP. In the end we obtain a distribution of all SNPs along the genome, with the relative distance between, therefore
# it can be seen how they are clustered.
p <- df.long %>%
    ggplot() +
    geom_text(aes(POS, strain, label = mutation1, color = SNP), fontface = "bold") +
    geom_vline(aes(xintercept = POS), alpha = 0.2, size = 0.2, data = df) +
    theme_bw() +
    theme(panel.grid = element_blank()) +
    scale_color_brewer(type = "qual", palette = "Set1")


## read and process gff file
gff <- fread("ref.gff") %>% tidyr::separate(V9, into = paste0("gff", 1:9), sep = ";", remove = FALSE)
# Below the graph, additional lines with information are added, first the position of rRNA genes as reference, and second the position of
# each gene, with its name and annotation according to NCBI.
p2 <-gff %>%
      dplyr::filter(V3 == "CDS" | V3 == "rRNA") %>%
      plot_ly() %>%
          add_segments(x = ~ V4, xend = ~ V5,
               y = ~ V3, yend = ~ V3,
               opacity = 0.7,
               color = ~ V7, size = I(14),
               colors = c("red", "forestgreen"),
               hoverinfo = "text",
               text =  ~paste("<b>", gff1, "</b>", "<br />", gff6))

## A function called "slideFunct" was created, it consists of a "window" which length can be set and it runs through every position
# along the genome, giving a score as a result. This score gets plotted and tells us if there are many or few SNPs clustered within the
# coverage of the window for a certain position.
# The slidefunct takes uses the formula: (1/(distance between the two closest SNPs within the window))x100
# For example here the window encompasses 5000 bp, and makes steps of 500 bp.
scores <- slideFunct(1:2501304, 5000, 500, df$POS)

p3 <- scores %>% plot_ly() %>% add_lines(x = ~ spots, y = ~ corrresult, color = I("red"))

#p4 <- scores %>% plot_ly() %>% add_lines(x = ~ spots, y = ~ res, color = I("blue"))

# In a fourth horizontal axe, the amount of mutants that contain a SNP per position is plotted in bars. Therefore, with both the
# cluster score and the number of SNPs in each position, it can be seen which are the most mutated areas which
p4 <- df.cons %>% plot_ly() %>% add_trace(x = ~ POS, y = ~ n, type = "bar", color = I("black"))

subplot(p, p2, p3, p4, shareX = TRUE, nrows = 4, heights = c(0.6, 0.1, 0.15, 0.15), titleX = FALSE) %>%
  layout(#width = "1200",
         #height = "600",
         showlegend = FALSE,
         yaxis = list(showgrid = FALSE,
                      showline = FALSE))
### Cheers!
```

Slide Function Script

```r
slideFunct <- function(data, window, step, snps){

   total <- length(data)

  spots <- seq(from = 1, to = (total - window), by = step)

  result <- vector(length = length(spots))

  corrresult <- vector(length = length(spots))
```

```
  for(i in 1:length(spots)){
    spotarray <- data[spots[i]:(spots[i]+window)] %in% snps

    # which(arr) gives the indices of TRUE values, from where we can get the distances
    # naive approach - sum of element-wise difference times number of TRUE?
    # 1/diff(which(arr))*length(arr) ????? will this work?

    result[i] <- sum(spotarray)
    if (length(which(spotarray)) <= 1) {
      corrresult[i] <- result[i]
    }else {
      corrresult[i] <- length(spotarray)/min(diff(which(spotarray)))
    }
  }
  return(data.table(spots = spots, res = result, corrresult = corrresult))
}
```

Table S1. M-mutants linkage assay results.

Mut N°	Total transformants	White CFU	Blue CFU	% linkage
**M43**	41	41	0	0.00%
**M6**	17	17	0	0.00%
**M16**	23	23	0	0.00%
**M92**	80	80	0	0.00%
**LacZ rep**	8000	8000	0	0.00%
**M94**	3124	3124	0	0.00%
**M4B**	25	25	0	0.00%
**M87**	672	672	0	0.00%
**M33**	5088	5088	0	0.00%
**M108**	20	20	0	0.00%
**M54**	3121	3120	1	0.03%
**M11**	2515	2512	3	0.12%
**M10**	1605	1600	5	0.31%
**M107**	27021	26870	151	0.56%
**M101**	4829	4800	29	0.60%
**M89**	2690	2672	18	0.67%
**M97**	3544	3520	24	0.68%
**M97**	3544	3520	24	0.68%
**M28**	558	554	4	0.72%
**M2**	1976	1960	16	0.81%
**M44**	5414	5360	54	1.00%
**M7**	1707	1688	19	1.11%
**M47**	6052	5984	68	1.12%
**M105**	3876	3832	44	1.14%
**M85**	4680	4624	56	1.20%
**M27**	5835	5760	75	1.29%
**M28B**	3890	3840	50	1.29%
**M1090**	10925	10784	140	1.29%
**M96**	6278	6192	86	1.37%
**M29**	5877	5760	117	1.99%
**M106**	6581	6400	181	2.75%
**M104**	47	30	17	36.17%
**M5**	2305	45	2260	98.05%
**C(-)**	0	0	0	-

Table S2. R-mutants linkage assay results

Mut N°	Total transf.	Blue	White	% linkage
**C(-)**	0	0	0	-
**LacZ**	5001	5000	1	0.02%
**R21**	4002	4000	2	0.05%
**R27**	5005	5000	5	0.10%
**R3**	1906	1904	2	0.10%
**R42**	4005	4000	5	0.12%
**R7**	793	792	1	0.13%
**R41**	3246	3240	6	0.18%
**R16**	1203	1200	3	0.25%
**R30**	4012	4000	12	0.30%
**R33**	350	348	2	0.57%
**RE**	1308	1300	8	0.61%
**R13**	5033	5000	33	0.66%
**R4**	4286	4256	30	0.70%
**R34**	1862	1840	22	1.18%
**R29**	80	80	1	1.25%
**R25**	2321	1	2320	99.96%
**R18**	5001	1	5000	99.98%
**R2**	5000	0	5000	100.00%
**R8**	428	0	428	100.00%
**R39**	2500	0	2500	100.00%
**R24**	2500	0	2500	100.00%
